# *Cytofast*: A workflow for visual and quantitative analysis of flow and mass cytometry data to discover immune signatures and correlations

**DOI:** 10.1016/j.csbj.2018.10.004

**Published:** 2018-10-24

**Authors:** Guillaume Beyrend, Koen Stam, Thomas Höllt, Ferry Ossendorp, Ramon Arens

**Affiliations:** a Department of Immunohematology and Blood Transfusion, Leiden University Medical Center, Albinusdreef 2, Leiden 2333 ZA, The Netherlands; b Department of Parasitology, Leiden University Medical Center, Leiden, The Netherlands; c Department of Computer Graphics and Visualization Group, Faculty of Electrical Engineering, Mathematics and Computer Science, Delft University of Technology, Delft, the Netherlands

**Keywords:** Flow and mass cytometry analysis, R-based package, High-dimensional analysis, Single-cell analysis, Visualization tool

## Abstract

Multi-parametric flow and mass cytometry allows exceptional high-resolution exploration of the cellular composition of the immune system. A large panel of computational tools have been developed to analyze the high-dimensional landscape of the data generated. Analysis frameworks such as FlowSOM or Cytosplore incorporate clustering and dimensionality reduction techniques and include algorithms allowing visualization of multi-parametric cytometric analysis. To additionally provide means to quantify specific cell clusters and correlations between samples, we developed an R-package, called *cytofast*, for further downstream analysis. Specifically, *cytofast* enables the visualization and quantification of cell clusters for an efficient discovery of cell populations associated with diseases or physiology. We used *cytofast* on mass and flow cytometry datasets based on the modulation of the immune system upon immunotherapy. With *cytofast,* we rapidly generated visual representations of group-related immune cell clusters and showed correlations with the immune system composition. We discovered macrophage subsets that significantly decrease upon cancer immunotherapy and distinct prime-boost effects of prophylactic vaccines on the myeloid compartment. *Cytofast* is a time-efficient tool for comprehensive cytometric analysis to reveal immune signatures and correlations. *Cytofast* is available at Bioconductor.

## Graphical abstract

**Figure:**
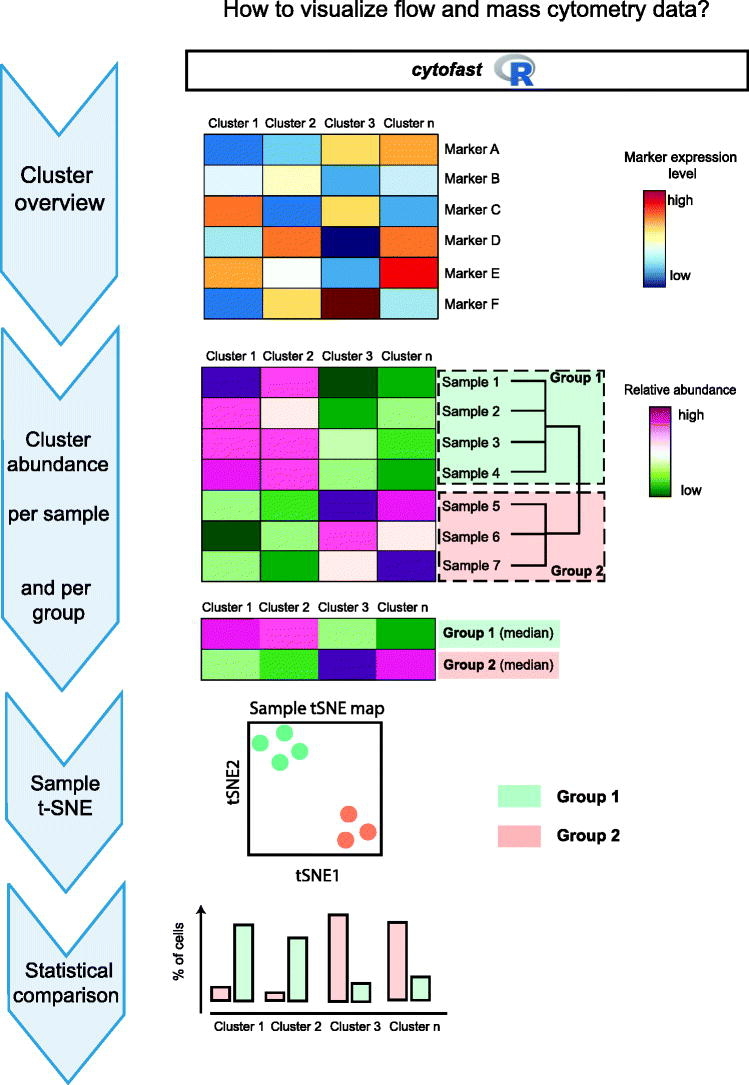

## 1 Introduction

Mass cytometry (cytometry by time-of-flight; CyTOF) can detect as many as 40 markers present on millions of single cells. The number of studies based on mass cytometry has considerably increased over the last few years, broadening simultaneously the choice of clustering techniques, such as SPADE [1], FlowMaps [2], FlowSOM [3], Phenograph [4], VorteX [5] and Scaffold maps [6], but also dimensionality reduction-based techniques including PCA [7], t-SNE [8] and Diffusion Map [9]. A recent new computational tool, HSNE [10], embedded in Cytosplore [11], proposed a combination of the two afore-mentioned techniques, building a hierarchical representation of the complete data that preserves the non-linear high-dimensional relationships between cells and avoids any down-sampling [12]. The aforementioned computational tools lack, however, the means for downstream analysis, where no automatic process has been proposed to link clusters abundance with e.g. clinical outcome and to visually interpret the data in the context of the experimental setup. Citrus [13] is currently a broadly used analysis tool for statistical comparison but presents several limitations. The number of samples to be included in such analysis should be more than eight and the number of cells are randomly downsampled before analysis. Moreover, Citrus is using agglomerative clustering, which brings complexity in data visualization.

Here, we developed a workflow, called *cytofast*, for a fast and quantitative analysis of flow and mass cytometry data. *Cytofast* allows the visualization of cluster phenotypes, their abundance per sample and per group and additionally enables statistical comparisons taking in account different clinical outcome variables.

We verified our workflow on a non-paired mass cytometry dataset originating from a published study focused on differences between effective and ineffective treatment. We also demonstrated the use of *cytofast* on a paired dataset of a prime-boost vaccination study. In addition, we verified *cytofast* on a flow cytometry dataset. Together these analyses showed that our workflow is valid, replicating similar findings previously described and in addition provided a deeper exploration of the data by newly identifying cell clusters that correlate to treatment.

## 2 Results and discussion

### 2.1 *Cytofast*: workflow presentation

We designed an R package, named *cytofast*, and introduced a workflow to quantify and identify significant group-related subsets. *Cytofast* can be used after cluster analysis (for example with Cytosplore or FlowSOM) has been performed. Here, we focused on the clustering analysis with Cytosplore using mass and flow cytometry datasets.

**Fig. 1:**
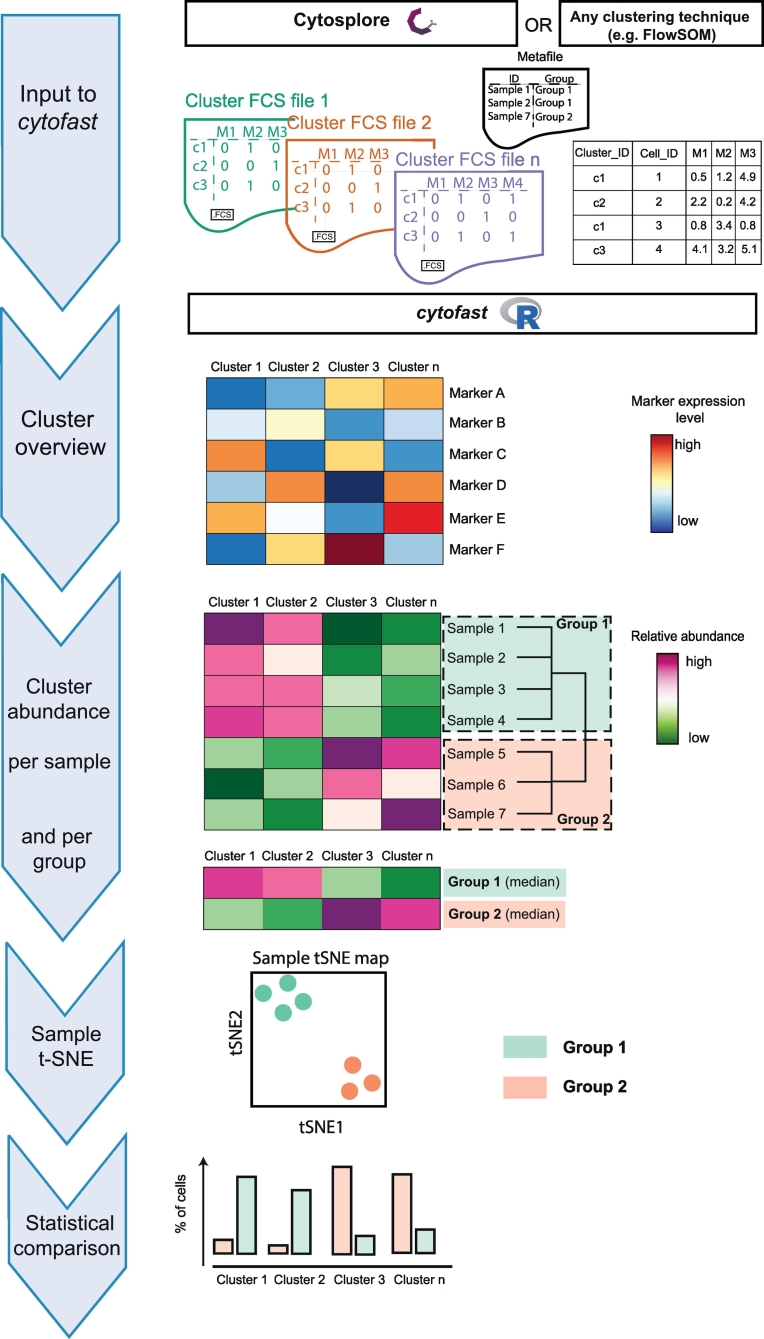
Schematic overview of the *cytofast* workflow. Flow and mass cytometry data processed by Cytosplore or other clustering techniques (e.g. FlowSOM) can be used as input for quantification and exploration of cell subset clusters. Cluster visualization, cluster abundance per sample and quantitative comparisons are automated and displayed in a user-friendly manner.

The workflow of *cytofast* can be divided in four parts (Fig. 1). First, a heatmap with a dendrogram is generated showing the median ArcSinh-transformed marker expression values (blue-to-red scale) for all the identified clusters (cluster phenotype overview). Second, a quantitative heatmap is generated showing the cell frequency calculated for each cluster stratified per individual sample. Thus, one row is representing one biological sample and the identified subsets are displayed per column (cluster abundance per sample). A dendrogram, represented on the side of the panel, indicates the clustering of the samples sharing phenotypic similarities. Hierarchical clustering was performed on subset frequencies using the Euclidean distance and complete linkage clustering. The summary of the quantitative heatmap can be displayed underneath taking in account the median abundance of each cluster per group. Next, a dimensionality reduction analysis based on cluster frequency is performed. As a result, a t-SNE map is drawn, where one dot is representing one sample colored by group assignment, proposing an alternative way to represent similarities between samples. Finally, the abundance of each cluster per group is represented in a quantitative bar graph. Statistical comparison is performed to highlight significant changes in cluster abundance between groups.

### 2.2 *Cytofast* applied to a non-paired mass cytometry datasets: comparison between effective and ineffective cancer immunotherapy

We tested our workflow on an unpaired mass cytometry data set from *Spitzer et al.* [14]. The authors characterized two effective therapies in mice by a combination of tumor-binding antibodies and adjuvants (B6-alloIgG + anti-CD40 + IFN-γ, CD-1-alloIgG + anti-CD40 + IFN-γ; together called *Eff*) and compared these to two ineffective therapies [15] (anti-PD-1, no treatment; together called *Ineff*). The experimental mouse model used was the spontaneous MMTV-PyMT (murine mammary tumor virus-polyoma middle T) model of breast cancer, which is refractory to other immunotherapies such as checkpoint blockade (i.e. anti-PD-1). At an early (Day 3) and later (Day 8) stage, mice were sacrificed and immune cells analyzed (n = 3–4 per treatment, per timepoint). We focused on comparing the effective and ineffective immune responses in the spleen at the two different timepoints. The data from the two effective and the two ineffective treatments were combined, resulting in one large effective treated group and one large ineffective treated group. Next, the data from the early and later timepoints of the effective and ineffective treated groups were pooled, resulting in four different groups. This approach allows then the simultaneous analysis of both treatment and time.

**Fig. 2:**
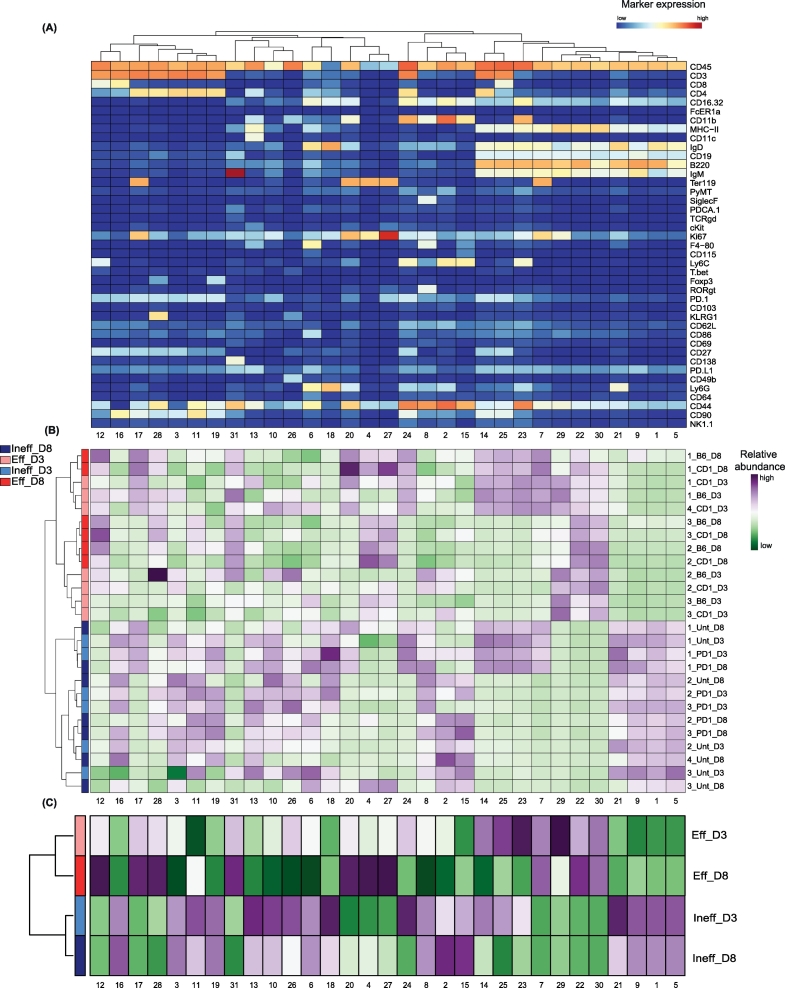
Identification and abundance of CD45^+^ cell clusters by *cytofast*. (A) Mass cytometry data of CD45^+^ immune cells in the spleen of mice that received effective and ineffective treatment at two different timepoints (Day 3 and Day 8). Heatmap of all 31 CD45^+^ cell clusters identified independent of treatment based on Cytosplore clustering. Level of ArcSinh5-transformed expression marker is displayed by a blue-to-red scale. Dendrogram on the top represents the hierarchical similarity between the identified clusters and is based on Euclidean distance and complete linkage clustering. (B) Heatmap of relative abundance (expressed as variance or dispersion from the mean) for each cluster identified above in each individual mouse. One row is representing one mouse sample, subsets are displayed per column. A green or a purple square is representative of a lower or a higher number of cells, respectively, compared to the average. The dendrogram displayed on the left is based on hierarchical clustering using Euclidean distance and complete linkage clustering. (C) Summary of the data represented on the panel above by using the median for each group.

Upon processing the data (containing 3.8 million cells) with Cytosplore, thirty-one clusters could be identified (Fig. 2A), and included distinct subsets of cytotoxic T cells (clusters 12, 16), helper T cells (e.g. clusters 17, 28), B cells (e.g. clusters 29, 22, 30), myeloid cells (e.g. cluster 21 identifying granulocytes) and erythroid lineages (clusters 4 and 27). The generated abundance heatmap for each individual sample quantitatively compared the abundance of each cluster between ineffective and effective groups or between an early (Day 3) and late (Day 8) response, (Fig. 2B). The two treatment groups (ineffective and effective) on the dendrogram of the heatmap could be distinguished. The summary heatmap, which displays the data based on the median of each group, is displayed to recapitulate the observed changes (Fig. 2C).

**Fig. 3:**
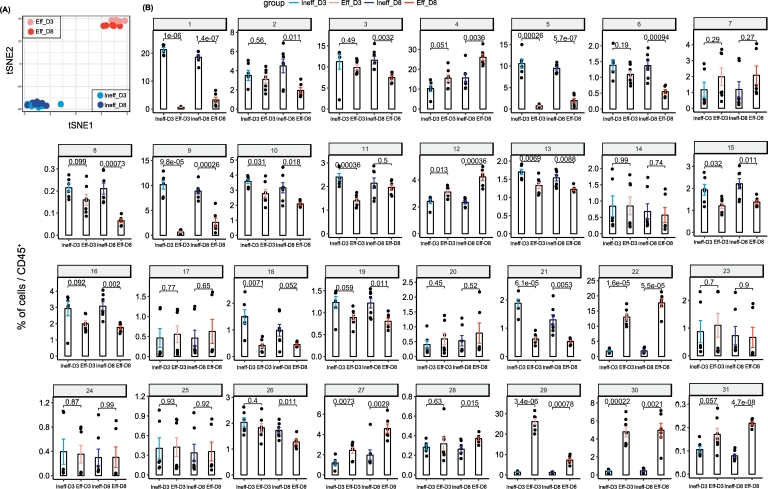
Quantitative comparison of cell clusters from unpaired samples by *cytofast*. (A) Sample t-SNE of the data of Fig. 2 where one dot is representing one sample, based on the cluster frequencies. The two groups, ineffective and effective, can be clearly distinguished, showing that the immune system is differently shaped by treatment efficiency. (B) Bar graphs representing the average of each sample per group overlaid with the dot plots representing the percentage of the cluster for each sample. P-values are provided to indicate significant differences between the ineffective and the effective group for both timepoints. Statistical analysis was performed on individual clusters (annotated from 1 to 31) using a t-test.

Based on the abundance of each cluster per sample, we established a sample-t-SNE map (Fig. 3A). We could reveal that the ineffective treated mice clustered together and that also the effective treated mice clustered, irrespective of the time-point of analysis. The absence of substantial differences on the immune system at an early stage (Day 3) or later stage (Day 8) indicates that the treatment effect is rapid and remains prevailing in time. We next analyzed the abundance of each cluster per group and per timepoint (Fig. 3B). We established that B cells expressing MHC class II (MHC-II) (clusters 29, 22 and 30) were more abundant upon effective treatment. Moreover, we newly identified two myeloid clusters (clusters 6 and 8), which were significantly reduced upon effective therapy, specifically at a later stage. These two subsets presented the highest expression in F4/80 among all the identified clusters (cluster 6: F4/80^+^/Ly6G^+^/CD44^+^/IgD^+^, cluster 8: F4/80^+^/CD11b^+^/Siglec-F^+^).

In conclusion, our workflow enables the overview of a large data set containing four different groups and improves the profiling of immune subsets that relate to therapy.

### 2.3 *Cytofast* applied to paired mass cytometry datasets: effect of prime-boost vaccination

To analyze paired samples by *cytofast*, we selected a partial dataset from *Palgen et al.* [16]. The mass cytometry data consists of blood immune cells analysed one day after first and second immunization of cynomolgus macaques with modified vaccinia virus Ankara (MVA).

**Fig. 4:**
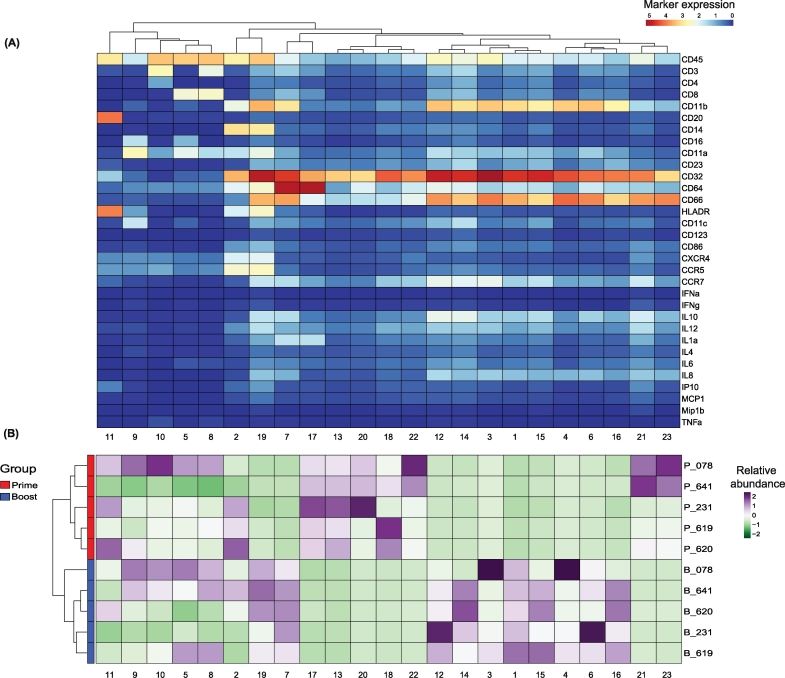
Identification and abundance of CD45^+^ cell clusters in the blood of cynomolgus macaques after prime and boost immunization with modified vaccinia virus Ankara. (A) Heatmap of all 23 CD45^+^ cell clusters identified independent of treatment based on Cytosplore clustering. Level of ArcSinh5-transformed expression marker is displayed by a blue-to-red scale. Dendrogram on the top represents the hierarchical similarity between the identified clusters. Dendrogram displayed above is based on hierarchical clustering using Euclidean distance and complete linkage clustering. (B) Heatmap of relative abundance (expressed as variance or dispersion from the mean) for each cluster identified above in each individual macaque. One row is representing one macaque blood sample, subsets are displayed per column. A green or a purple square is representative of respectively a lower or a higher number of cells compared to the average. Dendrogram displayed on the left is based on hierarchical clustering using Euclidean distance and complete linkage clustering.

The clustering analysis from Cytosplore identified twenty-three clusters, whose phenotype were presented by the heatmap (Fig. 4A). The abundance of each cluster stratified per sample showed a clear distinction between the immune response after prime and boost (Fig. 4B).

**Fig. 5:**
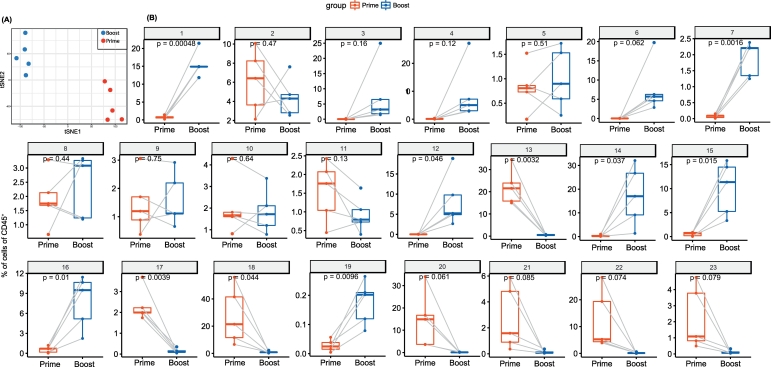
Quantitative comparison of cell clusters from paired samples by *cytofast*. (A) Sample t-SNE where one dot is representing one sample, based on the sample frequencies. The two groups can be clearly distinguished, showing that the immune system is differently shaped upon the two immunizations. (B) Box plots representing the average and standard deviation of each sample per group. Samples are paired. P-values are provided to indicate the significance between different groups. Statistical analysis was performed for each individual cluster (annotated from 1 to 23) using a t-test.

The difference between the two groups are clearly represented by the sample t-SNE map (Fig. 5A). The two populations after prime and boost are distinct, and do not cluster per individual, revealing that the variation triggered on the immune system of each individual is stronger than their intrinsic immune signature. The average abundance of each cluster per group can be displayed in bar graphs, and such quantification of the data allows thus direct comparisons between groups. We choose to characterize here the difference between the two groups by paired analysis for each identified cluster (Fig. 5B). After prime, certain cell subsets (characterized by CCR7^+^, IL-8^+^ and IL-10^+^) were absent but these were specifically detected after boost (clusters 1, 7, 12, 14, 15, 16). Conversely, other clusters, displaying low cytokine levels like IL-10, IL-8 or the CCR7 receptor were only identified after prime but absent after boost (clusters 17, 18, 20, 21, 22, 23).

### 2.4 *Cytofast* applied to flow cytometry data

With proper adjustment of the clustering in Cytosplore, *cytofast* can also be applied to flow cytometry datasets. We tested here a flow cytometry dataset comparing phenotypes of blood cells between patients suffering from acute myeloid leukemia (AML) and non-affected patients [17] (downloaded from FlowRepository [18]). After processing six random samples from each group (non-affected patients called *Normal*, and affected patients called *AML*), we applied *cytofast* and obtained a similar visualization output to mass cytometry data (Fig. S1). Thus, *cytofast* is also operational for visualization of complex flow cytometry data sets and can rapidly identify immune subset changes between groups.

## 3 Conclusion

Here we report the helpfulness of an R-based workflow, named *cytofast*, which is designed for visual and quantitative analysis of flow and mass cytometry data to discover immune signatures and correlations. We used a non-paired dataset generated by Spitzer and colleagues in which the effects of immunotherapy were examined, and newly identified with *cytofast* two macrophage clusters (F4/80^+^/Ly6G^+^/CD44^+^/ IgD^+^ and F4/80^+^/ MHC-II^+^/ Siglec-F^+^) that were significantly reduced upon effective therapy, specifically at a later stage. In a prophylactic vaccine study by Palgen and colleagues, paired samples were analysed and showed clear booster effects of the vaccine on myeloid cell subsets. The usefulness of *cytofast* to distinguish different immune signatures between groups was also observed with flow cytometry datasets of immune cells in the blood of leukemia patients and non-affected individuals.

Some of the displayed clusters appear highly similar in the heatmaps. It is optional for the user to merge similar clusters. Such cluster merging might remove subtleties, yet this might also make the results more robust against individual sample variation or batch effects.

For all tested datasets, *cytofast* yielded rapid results. With standard modern computer hardware and specifications (32GB RAM memory), the output was produced in less than thirty seconds (excluding clustering analysis with e.g. Cytosplore), making this tool highly suitable for a rapid and visual data screening.

## 4 Material and methods

### 4.1 Use of clustering method

The two cohort mass cytometry datasets were downloaded from *Cytobank.*

#### 4.1.1 Use of cytosplore

The files were uploaded to Cytosplore, already pre-gated by the original authors. Files were sample-tagged by adding the CSPRL_ST channel, their marker expression arcsinh5 transformed and subjected to dimensionality reduction. The iterations chosen of the HSNE analysis were 1,000. We clustered the data with a kernel size sigma of 30 on the overview level and exported the resulting clusters without manually modifying it. Lastly, in R we used *cytofast* for further analysis of those files.

#### 4.1.2 Use of other clustering method

The use of *cytofast* is not restricted to a pre-processing of the data with Cytosplore. Most of the clustering providing a cluster assignment to each cell can be used (e.g. FlowSOM as detailed in the *vignette*).

### 4.2 Use of *cytofast*

Our workflow is linked to the use of an upstream clustering tools such as Cytosplore or FlowSOM. We load the clusters produced by applying the HSNE dimensionality reductions and mean shift clustering in Cytosplore and saved as FCS files. After loading the FCS files of all clusters, *cytofast* analysis is based on the sample characteristics written to a single file, gathering relevant information like sample name, clinical outcome and sample tag (also named CSPRL_ST in Cytosplore). The clinical input can be any qualitative clinical data like gender, or group affiliation, or quantitative like tumor size or age.

The *vignette*, where all the steps are explained to facilitate the reproducibility of the figures and the package itself will be deposited on Bioconductor. A downsampled number of cells from the *Spitzer* study is included in the vignette. Differences might be seen between the downsampled dataset provided in the vignette and the analysis from this paper due to the downsampling.

### 4.3 Flow cytometry data

We confirmed that our R script was also valid to represent flow cytometry datasets. We downloaded data files from FlowRepository (https://flowrepository.org/id/FR-FCM-ZZYA) and selected six patients per group (Files 0006.FCS, 0014.FCS, 0022.FCS, 0030,FCS, 0046.FCS, 0062.FCS from the *Normal* group; Files 0038.FCS, 0070.FCS, 0206.FCS, 0262.FCS, 0294.FCS, 0390.FCS from the *AML* group). Data was clustered with Cytosplore, clusters sharing high similarities were manually merged and the resulted output were processed with *cytofast*.

## Author contributions

GB wrote the manuscript and the workflow. KS wrote and embedded the workflow into the R package *cytofast*. TH reviewed the manuscript and gave conceptual input. FO gave conceptual input. RA wrote the manuscript and supervised the project.

## Conflict of interest

The authors declare no financial or commercial conflict of interest.

## Appendix A Supplementary data

Supplementary Figure S1 Image 1
