# Supplementary material for: Cytofast: A workflow for visual and quantitative analysis of flow and mass cytometry data to discover immune signatures and correlations
Source: Comput Struct Biotechnol J. 2018 Oct 24;16:435–42. doi: 10.1016/j.csbj.2018.10.004 (PMC6226576; doi:10.1016/j.csbj.2018.10.004)
Supplement: Supplementary file 1 — Supplementary Figure S1 [file mmc1.docx]

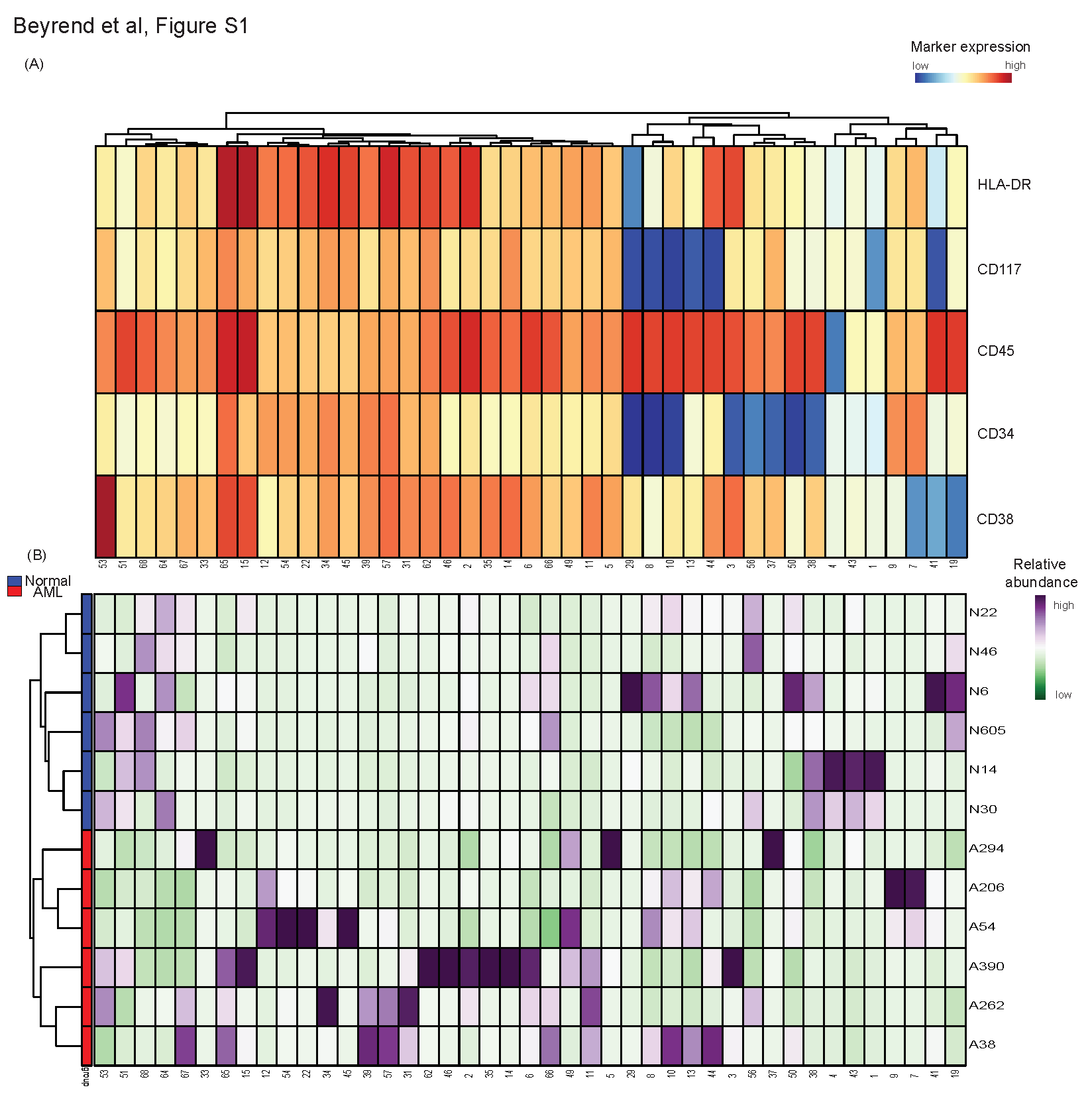


**Figure S1.** **Application of *cytofast* to flow cytometry data of patient samples**

*(A) Heatmap of all 43 CD45^+^ cell clusters identified independent of disease status on Cytosplore clustering. Level of ArcSinh25-transformed expression marker is displayed by a blue-to-red scale. Dendrogram on the top represents the hierarchical similarity between the identified clusters and is based on hierarchical clustering using Euclidean distance and complete linkage clustering.*

*(B) Heatmap of relative abundance (expressed as variance or dispersion from the mean) for each cluster identified above in each individual. One row is representing one patient blood sample, subsets are displayed per column. A green or a purple square is representative of respectively a lower or a higher number of cells compared to the average. Dendrogram displayed on the left is based on hierarchical clustering using Euclidean distance and complete linkage clustering.*
